# Characterization of Bioactive Compounds in *Elaeagnus conferta* Leaf Extract Using UPLC‐Q‐TOF‐MS and Evaluation of Its Anti‐Obesity Effects

**DOI:** 10.1002/fsn3.70226

**Published:** 2025-05-25

**Authors:** Jiali Zhang, Peicong Wu, Yingdui He, Xiaoping Zang, Hongbin Yang, Mamdouh A. Eissa, Yanxia Li, Weihong Ma, Ming Feng, Jiashui Wang

**Affiliations:** ^1^ Tropical Crops Genetic Resources Institute, Chinese Academy of Tropical Agricultural Sciences; Key Laboratory of Crop Gene Resources and Germplasm Enhancement in Southern China, Ministry of Agriculture; Key Laboratory of Tropical Crops Germplasm Resources Genetic Improvement and Innovation of Hainan Province Haikou China; ^2^ Haikou Experimental Station Chinese Academy of Tropical Agricultural Sciences Haikou China; ^3^ Institute of Tropical Bioscience and Biotechnology, Chinese Academy of Tropical Agricultural Sciences, Key Laboratory of Genetic Improvement of Bananas, Sanya Research Institute, State Key Laboratory of Biological Breeding for Tropical Crops Haikou Hainan Province China; ^4^ Department of Soils and Water Faculty of Agriculture, Assiut University Assiut Egypt

**Keywords:** bioactive compounds flavones, micro‐morphological, phenolic acids, subtropical shrub

## Abstract

This study aimed to characterize the bioactive compounds in *Elaeagnus conferta* Roxb. leaf extract using UPLC‐QTOF‐MS and subsequently assess its anti‐obesity effects in a murine model. The methanolic extract of *Elaeagnus conferta* leaves was analyzed, identifying 13 bioactive compounds, including flavones (rutin, kaempferol 3‐neohesperidoside, and spinacetin 3‐gentiobioside) and phenolic acids (caffeic acid 3‐O‐glucuronide and 1‐O‐feruloylglucose), known for their potential anti‐obesity properties. In the in vivo study, male C57BL/6J mice were fed a high‐fat diet (HFD) and supplemented with *E. conferta* extract at doses of 150 mg/kg/day and 300 mg/kg/day for 8 weeks. The administration of *E. conferta* leaf extract significantly reduced body weight gain in a dose‐dependent manner, with the highest reduction observed at 300 mg/kg/day. The extract also lowered low‐density lipoprotein cholesterol (LDL‐c) and total cholesterol (TC) while increasing brown adipose tissue (BAT) formation. Histological analysis revealed a reduction in adipocyte size and lipid accumulation in liver tissues and white adipose tissues, indicating improved fat metabolism. Moreover, *E. conferta* extract supplementation led to a significant decrease in intra‐abdominal and epididymal white adipose tissue weights compared to the HFD group. These findings suggest that *E. conferta* leaf extract exerts anti‐obesity effects by modulating lipid metabolism and adipose tissue distribution. This study provides a scientific basis for the potential application of *E. conferta* as a functional food ingredient for obesity management.

## Graphical abstract

Methanol extract from leaves of *Elatagnus conferta* was used as an anti‐obesity agent. UPLC‐Q‐TOF‐MS/MS showed that the extract contained 13 different chemicals. The extract contains eight flavones and four phenolic acids. The extract caused a significant reduction in the body weights of the mice under study.

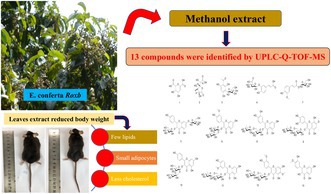

## 1 Introduction

*Elaeagnus conferta* Roxb. (Elaeagnaceae), commonly known as Yang Nai Guo in Chinese, is a native plant of South China, Vietnam, Malaysia, and India (Selvakumar et al. 2022; Zhuang et al. 2021) and is widely found in Yunnan, Guizhou, and Guangxi provinces of China (Cao et al. 2001; Fu and Wang 2007). It is a berry‐producing shrub with significant nutritional value. Species within the *Elaeagnus* genus are known for their medicinal properties, including reducing blood sugar, lowering lipid levels, antioxidative, anti‐inflammatory, and boosting immunity (Gupta et al. 2021; Selvakumar et al. 2022). The bioactive components of this genus include volatile oils, flavonoids, triterpenoids, alkaloids, steroids, anthraquinones, and fatty acids, with flavonoids being the most extensively reported (Bendaikha et al. 2014; Gupta et al. 2021; Liao et al. 2013; Lu et al. 2015; Selvakumar et al. 2022). While previous studies have focused on the nutritional composition of *E. conferta* fruit, including protein, fat, total sugar, crude fiber, tannins, and organic acids (Bendaikha et al. 2014; Liao et al. 2013; Lu et al. 2015; Selvakumar et al. 2022), limited research has been conducted on the bioactive components of its leaves (Ge et al. 2017; Gupta et al. 2021; Hu et al. 2014; Li and Shao 2019; Wu et al. 2011; Zhang and Liu 2021). Recently, the methanolic extract of *E. conferta* seeds was reported to possess anti‐ulcerogenic properties (Gupta et al. 2021), suggesting potential therapeutic applications. However, a comprehensive characterization of the bioactive constituents of *E. conferta* leaves remains largely unexplored (Guo et al. 2023; Gupta et al. 2021). Employing advanced analytical techniques can aid in the identification of essential bioactive compounds contributing to its pharmacological properties. To address this gap, we utilized Ultra‐Performance Liquid Chromatography‐Quadrupole Time of Flight Mass Spectrometry (UPLC‐QTOF‐MS), a state‐of‐the‐art analytical technique known for its rapid separation, high sensitivity, and precise mass accuracy (Guo et al. 2023; Sun et al. 2019; Yan et al. 2015). This method enables the identification of secondary metabolites by providing accurate molecular weight data and structural information, making it ideal for the chemical profiling of complex plant extracts (Zeng et al. 2022).

The increasing prevalence of obesity, a major global health concern, is closely linked to metabolic disorders such as diabetes, cardiovascular disease, and non‐alcoholic fatty liver disease (Mǎrginean et al. 2018; Salehi et al. 2019; Ulpathakumbura et al. 2023). The World Health Organization (WHO) reports that approximately 1.03 billion individuals worldwide are affected by obesity (Laurence 2023). This condition is characterized by excessive adipose tissue accumulation resulting from an imbalance between caloric intake and energy expenditure (Mǎrginean et al. 2018). Consumption of high‐fat and sugar‐rich diets contributes to obesity‐related complications, including cardiovascular disease, dyslipidemia, atherosclerosis, fatty liver, and hypertension (Laurence 2023). Although pharmacological treatments such as orlistat are available, they are associated with undesirable gastrointestinal side effects, limiting their long‐term use (Bendaikha et al. 2014). As a result, there is growing interest in natural bioactive compounds as alternative therapeutic agents for obesity management (Nderitu et al. 2023; Rani et al. 2012; Zheng et al. 2017).

Natural plant extracts, rich in flavonoids and phenolic acids, have been identified as promising anti‐obesity agents due to their ability to modulate lipid metabolism, reduce adipogenesis, and promote energy expenditure (Burcelin et al. 2002; Nderitu et al. 2023; Rani et al. 2012; Salehi et al. 2019; Zheng et al. 2017).

In this study, we employed a methanolic extraction method for *E. conferta* leaves due to its high efficiency in isolating flavonoids and phenolic acids, which are known contributors to anti‐obesity effects (Gupta et al. 2021). The extraction was followed by UPLC/Q‐TOF‐MS analysis to characterize the bioactive compounds (Guo et al. 2023), and the anti‐obesity potential of *E. conferta* extracts was evaluated using an in vivo C57BL/6J murine model. This model is widely used for obesity research due to its metabolic similarities with human obesity when subjected to a high‐fat diet (HFD) (Burcelin et al. 2002).

The study aimed to determine whether the identified compounds contribute to weight reduction, improved lipid metabolism, and regulation of adipose tissue. The objectives were first to characterize the bioactive components in *E. conferta* leaves and then to assess their anti‐obesity potential. This study provides a scientific basis for the potential application of *E. conferta* as a functional food ingredient for obesity management.

## 2 Materials and Methods

### 2.1 Materials

Methanol and acetonitrile of UPLC grade were purchased from Merck (Darmstadt, Germany), while formic acid (HPLC grade) was obtained from Tedia (Fairfield, USA). A Milli‐Q system (Millipore, France) was used to purify the water for the UPLC process. Jiashui Wang from the Chinese Academy of Tropical Agricultural Sciences authenticated the *Elaeagnus conferta* Roxb. leaves collected in July 2021 in Hainan Province, China, and the voucher specimens (No. 20210712) were deposited in the Key Laboratory of Crop Gene Resources, Ministry of Agriculture, People's Republic of China, in Haikou, Hainan. The plant sample was collected from the same species. Sniff Spezialdiäten GmbH, Soest, Germany, supplied the standard chow diet (normal‐fat diet (NFD)) (E15748‐04) and the HFD (E15744‐34). The HFD was a strict, lard‐based diet that provided energy in the following proportions: 20 kJ% from protein, 35 kJ% from carbohydrates, and 45 kJ% from fat. In contrast, the NFD was a precisely formulated control diet with the same nutrients as the HFD, differing only in the relative quantities of fat and carbohydrates, providing 13 kJ% from fat, 20 kJ% from protein, and 67 kJ% from carbohydrates.

### 2.2 Preparation of Plant Extracts

The leaves of *E. conferta* were collected and processed for extraction. The plant material was oven‐dried at 70°C for 48 h to ensure thorough dehydration and prevent microbial contamination. The choice of this drying temperature was based on previous studies indicating that moderate drying temperatures (60°C–80°C) effectively preserve bioactive compounds while minimizing enzymatic degradation and microbial growth (Tiho et al. 2017). Moreover, flavonoids and phenolic acids, which are the key bioactive constituents of *E. conferta* , are stable within this temperature range (Chen et al. 2011; Hamrouni‐Sellami et al. 2013).

The dried leaves were powdered, and 0.4 g of the sample was placed into a 5‐mL centrifugal tube for UPLC‐Q‐TOF‐MS/MS analysis. Methanol (4 mL) was added, and the mixture was ultrasonicated (42 kHz, 135 W; Branson Ultrasonic Corporation, USA) at room temperature for 30 min to extract compounds. The extract was filtered through a 0.45 μm filter, and 1 μL of the filtrate was injected into the UPLC‐Q‐TOF‐MS for chemical analysis.

For the animal study, a large‐scale extraction was performed using 2.0 kg of dried *E. conferta* leaf powder. The material was extracted in methanol under ultrasonic conditions (10:1 solvent to material ratio) at room temperature. The extract was filtered, concentrated, and freeze‐dried to obtain the final powdered leaf extract (referred to as YNG). The use of methanol as a solvent was justified by its high efficiency in extracting flavonoids and phenolic acids, which are responsible for the extract's anti‐obesity effects (Gupta et al. 2021; Wu et al. 2020). Methanol levels were below the permissible regulatory threshold of 3000 ppm (0.3%), as specified by the Guidelines on Residual Solvents (International Council for Harmonisation of Technical Requirements for Pharmaceuticals for Human Use (ICH) 2021). The extracts were reconstituted in 0.9% normal saline before oral gavage to ensure complete elimination of any risk of methanol exposure to the animals.

### 2.3 UPLC‐Q‐TOF‐MS Analysis

Waters ACQUITY UPLC system with an ACQUITY UPLC HSS T3 C18 column (2.1 × 100 mm 1.8 μm, Waters, Milford, MA, USA) was used in the UPLC‐Q‐TOF‐MS analysis. MS data was obtained from Waters Xevo G2 QTOF (Waters, Milford, MA, USA) equipped with a Z‐Spray ESI source. One microliter (1 μL) of each sample solution was injected into a solvent system at a 0.3 mL/min flow rate. The solvent system contained A: 0.1% aqueous formic acid and B: acetonitrile with 0.01% formic acid. The elution conditions were as follows: 0–1 min, 6%–18% B; 1–2 min, 18%–20% B; 2–6 min, 20%–22% B; 6–9 min, 22%–30% B; 9–12 min, 30%–36% B; 12–16 min, 36%–99% B; 16–19 min, 99%–99% B; 19–20 min, 99%–6% B; and 20–23 min, 6%–6% B. The autosampler's and column's temperatures were kept at 10°C and 40°C, respectively. The specific operation steps were similar to our reference report (Wang et al. 2020). The web databases ChemSpider Library, PubMed, MassBank, PubChem, and Natural Chemistry were used to identify the chemical compounds.

### 2.4 Animal Experiment

#### 2.4.1 Animal Model and Housing Conditions

Male mice (C57BL/6J, 6–8 weeks old) were kept in polycarbonate cages with four animals per cage. Cages were set up in an animal room with a 12‐h light/dark cycle, 20°C, and a relative humidity of 40%–50%.

#### 2.4.2 Diet and Groups

Mice were randomly assigned to four dietary groups (*n* = 6 per group) following a 1 week acclimatization period.

- NFD: standard chow diet.
- HFD 45% kCal from fat.
- HFD supplemented with *E. conferta* extract (150 mg/kg/day) (YNG‐L).
- HFD supplemented with *E. conferta* extract (300 mg/kg/day) (YNG‐H).

The YNG‐L and YNG‐H groups received intragastric administration of *E. conferta* leaf extract for 8 weeks. To avoid methanol exposure, the extracts were reconstituted in 0.9% normal saline before oral gavage, while the NFD and HFD groups administered only 0.9% normal saline via the intragastric route. These doses were chosen in reference to previous studies on flavonoid‐rich plant extracts with anti‐obesity potential (Gupta et al. 2021; Munkong et al. 2024; Wang et al. 2020; Wu et al. 2011). Then the chosen dosage (150 mg/kg/day and 300 mg/kg/day) was used in a preliminary pilot study to confirm nontoxicity as indicated by stable liver and kidney function markers in serum biochemistry tests (data not shown).

#### 2.4.3 Sample Collection

After 8 weeks, each mouse's body weight was noted. White adipose tissue (WAT), brown adipose tissue (BAT), and organs were taken after the mice were given 3 mL/kg of chloral hydrate to anesthetize them. Blood samples were centrifuged at 2000 ***g*** for 15 min at 4°C and then frozen at −80°C. Adipose tissue and organs were cleaned, weighed, frozen in liquid nitrogen, and maintained at −80°C.

#### 2.4.4 Biochemical Analysis

An automatic biochemical analyzer (Hitachi 7080, Japan) was used to determine total cholesterol (TC), triglycerides (TGs), high‐density lipoprotein cholesterol (HDL‐c), and low‐density lipoprotein cholesterol (LDL‐c) in serum.

#### 2.4.5 Histological Assessment

Liver tissues, intra‐abdominal white adipose tissue (IWAT), epididymal white adipose tissue (EWAT), and BAT were fixed in 4% paraformaldehyde and then embedded in paraffin. To assess histological steatosis, paraffin sections (5 μm) were stained with hematoxylin and eosin stain. The histological steatosis was analyzed at 100× magnification using a microscope (MOTIC AE31, Motic China Group CO, LTD, China).

#### 2.4.6 Ethical Considerations

Animal procedures were performed according to the Guidelines for Animal Care and Use Committee of the Chinese Academy of Tropical Agricultural Sciences (ACUCC), Hainan, PR China.

### 2.5 Statistical Analysis

The results are presented as mean ± SD. ANOVA analysis was conducted using GraphPad Prism 7.0 software. The Student's *t*‐test and one‐way ANOVA were used for single and multiple comparisons. A *p*‐value of less than 0.05 was considered statistically significant.

## 3 Results

### 3.1 UPLC‐Q‐TOF‐MS/MS Analysis of the Methanolic Extract of *E. conferta* Roxb Leaves

The mass spectrometry analysis of the methanolic extract of *E. conferta* leaves, conducted using UPLC‐Q‐TOF‐MS/MS in negative ionization mode, enabled the detection and identification of 13 secondary metabolites, comprising eight flavones, four phenolic acids, and one sugar derivative (Table 1). The negative ionization mode exhibited superior ionization efficiency and sensitivity, particularly for flavones, compared to the positive mode.

**TABLE 1:** The mass spectrometry data for the identification of chemical constituents of methanolic extract of *E. conferta* in negative ionization mode.

Peak No.	RTe (min)	Molecular formula	Molecular weight		*m/z* [M‐H]^−^		Mass error (ppm)	Identification
			Observed	Theoretical	Quasi‐molecular ion	Ion		
1	1.73	C_7_H_8_O_7_	204.0	204.0270	203.0201	—	1.7	Daucic acid
2	1.76	C_6_H_8_O_7_	192.0274	192.0270	191.0201	111.0089	1.9	D‐Glucaro‐1,4‐lactone
3	2.63	C_14_H_18_O_9_	330.0952	330.0951	329.0879	167.0352	0.2	3′‐Glucosyl‐2′,4′,6′‐trihydroxy acetophenone
4	2.70	C_15_H_16_O_10_	356.0745	356.0743	355.0673	191.0199	0.5	Caffeic acid 3‐*O*‐glucuronide
5	2.86	C_27_H_32_O_16_	612.1685	612.1690	611.1612	—	−0.9	Aromadendrin 3,7‐diglucoside
6	3.40	C_28_H_32_O_15_	608.1728	608.1741	607.1655	299.0555	−2.2	Calendoflaside
7	3.55	C_16_H_20_O_9_	356.1101	356.1107	355.1028	193.0499 175.0397	−1.7	1‐*O*‐Feruloylglucose
8	4.84	C_27_H_30_O_16_	610.1538	610.1534	609.1465	300.0279	0.7	Rutin
9	4.99	C_28_H_32_O_17_	640.1640	640.1639	639.1567	—	0.0	Ranupenin 3‐rutinoside
10	5.55	C_27_H_30_O_15_	594.1593	594.1585	593.1520	285.0405	1.4	Kaempferol 3‐neohesperidoside
11	5.72	C_28_H_32_O_16_	624.1697	624.1690	623.1624	315.0512	1.0	Keioside
12	5.73	C_29_H_34_O_18_	670.1748	670.1745	669.1675	315.0512	0.4	Spinacetin 3‐gentiobioside
13	6.98	C_16_H_14_O_7_	318.0742	318.0740	317.0669	152.0113 125.0243	0.7	Tamarixetin

**FIGURE 1:**
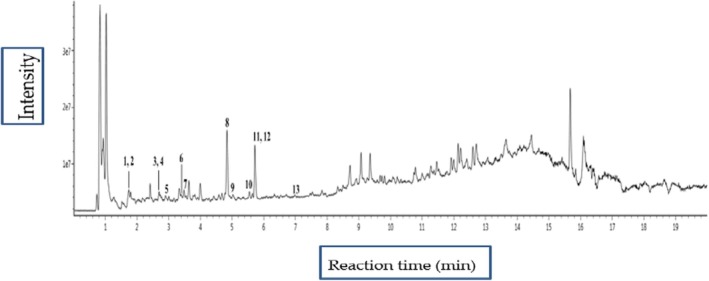
Total ion chromatograms of *E. conferta* *leaves* extract based on UPLC‐ESI‐QTOF‐MS/MS. Peak numbers are consistent with Table 1.

Figure 1 presents the total ion chromatogram, illustrating the retention times and intensities of the detected compounds. Each peak corresponds to a specific metabolite, with peaks 1, 2, 4, and 6 representing phenolic acids; peaks 3, 5, 8, 9, 10, 11, 12, and 13 corresponding to flavones, and peak 7 denoting the sugar derivative. The precise mass data and retention times of the metabolites were used to confirm their identities, as listed in Table 1.

**FIGURE 2:**
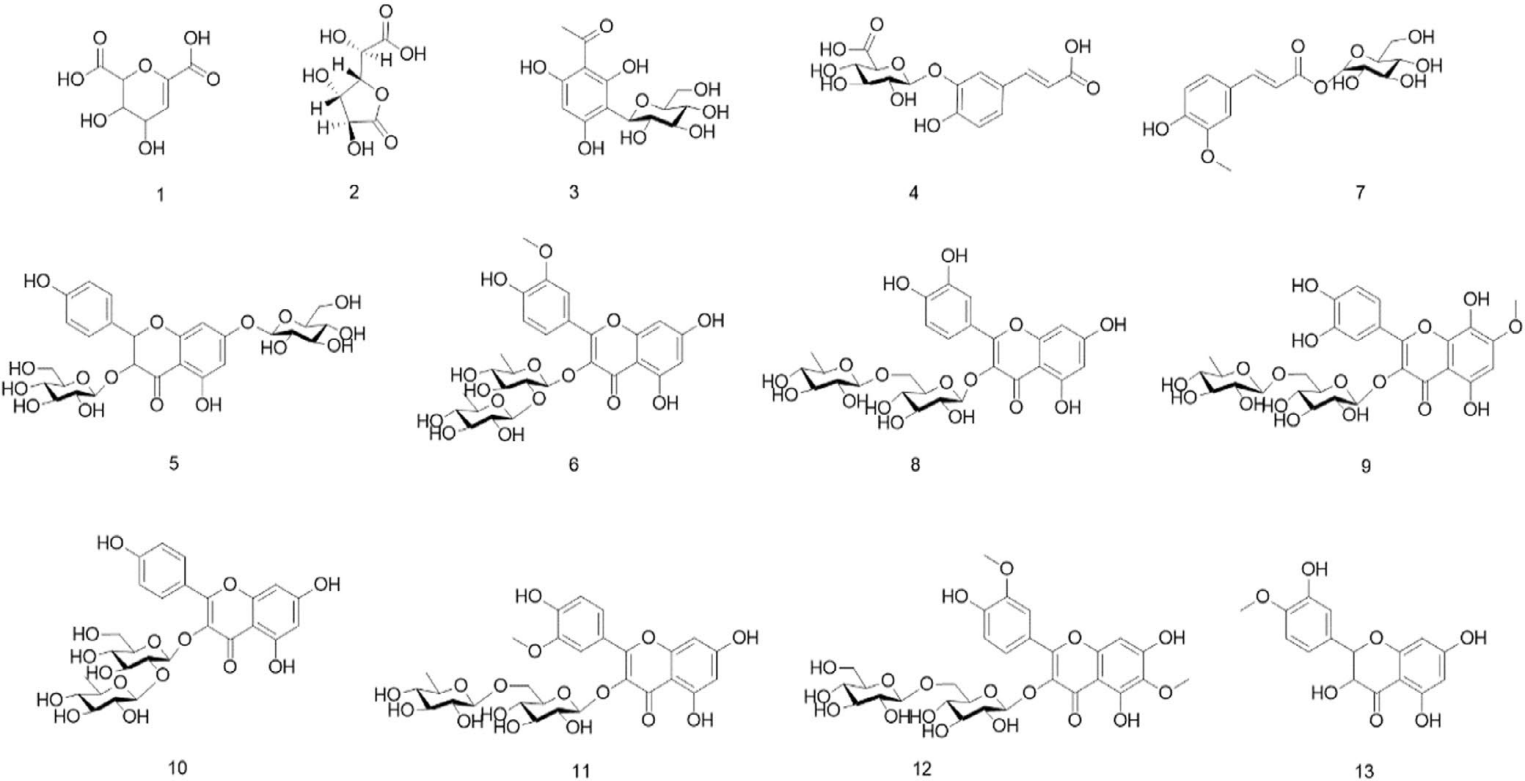
Structures of the compound identified from UPLC‐Q‐TOF‐MS/MS. The chemicals list is consistent with the peak number in Table 1.

Figure 2 displays the chemical structures of the 13 identified compounds, including both flavones and phenolic acids, which were validated through ESI‐MS/MS analysis. This comprehensive characterization highlights the chemical diversity of *E. conferta* leaves, with flavones being the predominant metabolites. The robust detection methodology employed in this study facilitates high‐resolution identification of these bioactive compounds, providing a foundation for further biological or pharmacological investigations.

### 3.2 In Vivo Anti‐Obesity Activity of *E. conferta* Leaves Extract

#### 3.2.1 Effects on Body Weight

**FIGURE 3:**
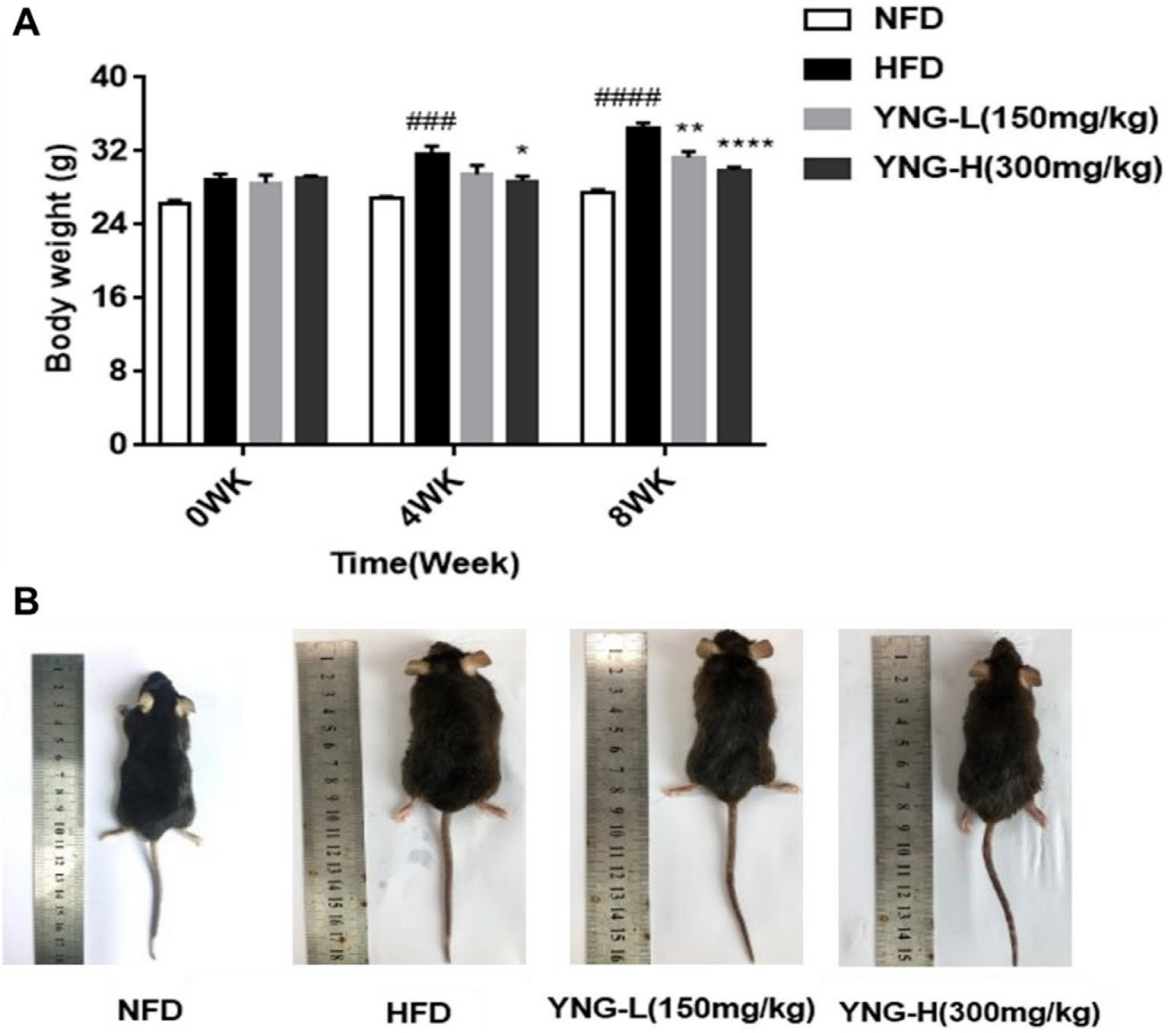
(A) Changes in body weight of mice after 0, 4, and 8 weeks of different diets. (B) Morphology of mice after 8 weeks of different diets. HFD, high‐fat diet; NFD, normal fat diet; YNG‐H, HFD receiving 300 mg/kg/day of *E. conferta* leaf extract; YNG‐L, HFD receiving 150 mg/kg/day of *E. conferta* leaf extract. Data are expressed as mean ± standard error (*n* = 6), *, **, and ****indicate significance at *p* < 0.05, *p* < 0.01, and *p* < 0.0001, respectively, compared with the HFD group; ^###^, and ^####^indicate significance at *p* < 0.001 and *p* < 0.0001, respectively, compared with the NFD group.

The anti‐obesity effect of *E. conferta* leaf extract was evaluated using a murine model. As illustrated in Figure 3A, mice fed on a HFD exhibited a significant increase in body weight after 4 and 8 weeks compared to those maintained on a NFD. In contrast, mice in the YNG‐H group (HFD supplemented with 300 mg/kg/day *E. conferta* leaf extract) demonstrated a marked reduction in body weight after 4 weeks of treatment. By week 8, both the YNG‐L (HFD receiving 150 mg/kg/day *E. conferta* leaf extract) and YNG‐H showed a significant weight reduction compared to the HFD group, with the YNG‐H group showing the most pronounced effect. Figure 3B confirms that mice receiving YNG‐L and YNG‐H supplementation over 8 weeks exhibited a substantial reduction in body weight, further supporting the potential of *E. conferta* in mitigating HFD‐induced obesity.

#### 3.2.2 Effects on Liver and Adipose Tissue

**FIGURE 4:**
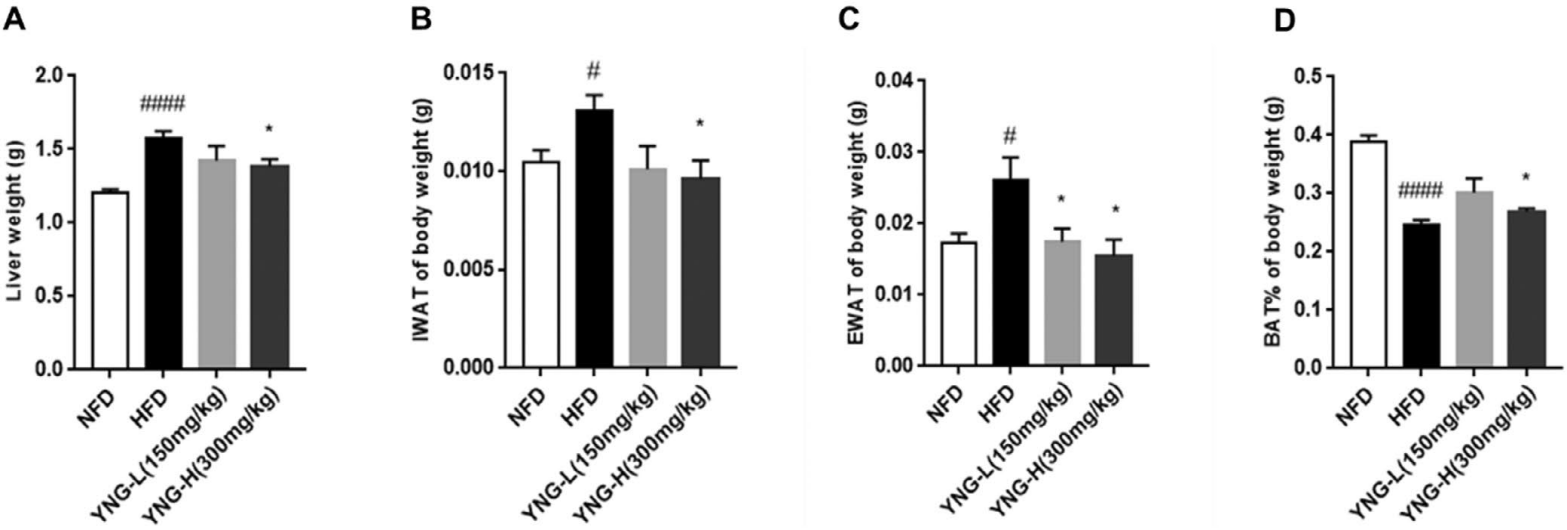
Effect of different diets on (A) liver weight, (B) intra‐abdominal white adipose tissue, (C) epididymal white adipose tissue, and (D) formation of brown adipose tissue of mice. BAT, brown adipose tissue; EWAT, epididymal white adipose tissue; HFD, high‐fat diet; IWAT, intra‐abdominal white adipose tissue; NFD, normal fat diet; YNG‐H, HFD receiving 300 mg/kg/day of *E. conferta* leaf extract; YNG‐L, HFD receiving 150 mg/kg/day of *E. conferta* leaf extract. Data are expressed as mean ± standard error (*n* = 6), *indicates significance at *p* < 0.05 compared with the HFD group; ^#^, and ^####^indicate significance at *p* < 0.05 and *p* < 0.0001, respectively, compared with the NFD group.

The liver weight of the HFD group was significantly higher than that of the NFD, indicating hepatic lipid accumulation (Figure 4A). However, YNG‐H supplementation effectively reduced liver weight compared to the HFD group, suggesting a protective effect against hepatic steatosis.

Furthermore, the HFD group exhibited a marked increase in IWAT, EWAT, and BAT compared to the NFD (Figure 4B–D). Notably, YNG‐H supplementation significantly reduced IWAT (Figure 4B) and BAT (Figure 4D) volumes, whereas both YNG‐L and YNG‐H treatments effectively decreased EWAT as compared to HFD (Figure 4C), suggesting a dose‐dependent impact on adipose tissue regulation.

#### 3.2.3 Effects on Serum Lipid Profile

**FIGURE 5:**
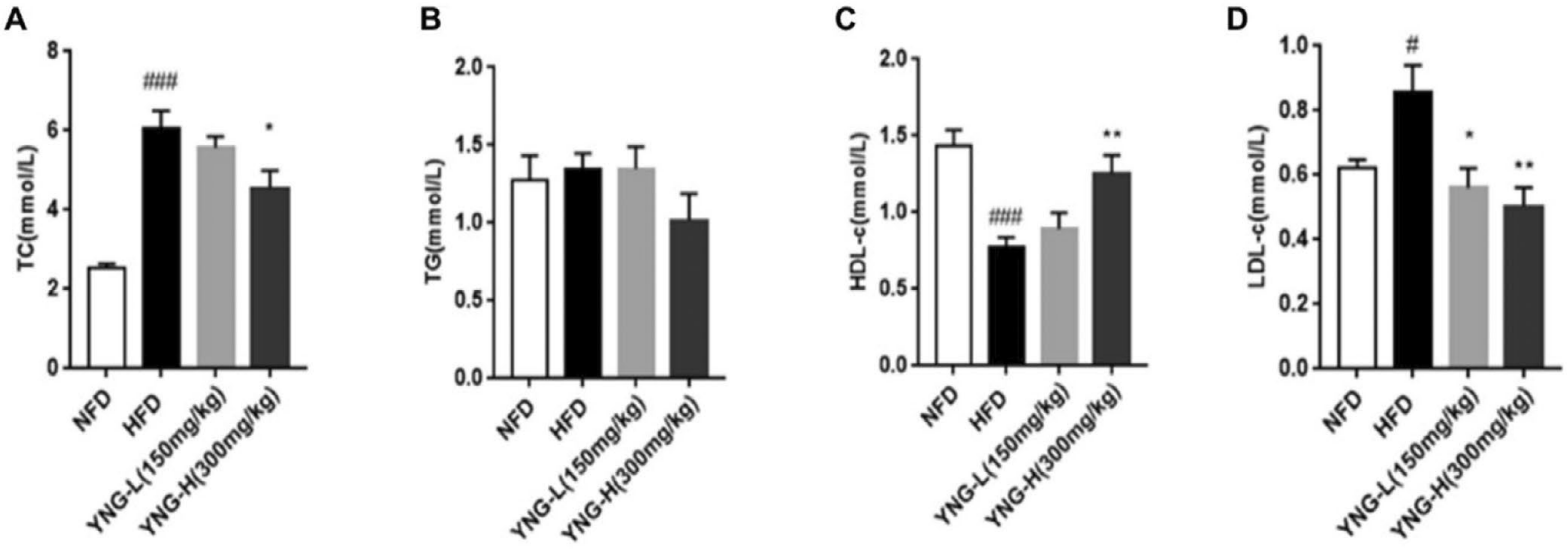
Effect of different diets on serum (A) total cholesterol, (B) triglycerides, (C) high‐density lipoprotein, and (D) low‐density lipoprotein of mice. HFD, high‐fat diet; NFD, normal fat diet; YNG‐H, HFD receiving 300 mg/kg/day of *E. conferta* leaf extract; YNG‐L, HFD receiving 150 mg/kg/day of *E. conferta* leaf extract. Data are expressed as mean ± standard error (*n* = 6). * and **indicate significance at *p* < 0.05 and *p* < 0.01, respectively, compared with the HFD group; ^#^, and ^####^indicate significance at *p* < 0.05 and *p* < 0.0001, respectively, compared with the NFD group.

HFD significantly increased the TC in serum compared to NFD (Figure 5A). However, YNG‐H supplementation significantly reduced TC levels compared to HFD, indicating a potential hypocholesterolemia effect. Conversely, TG levels remained unaffected by any treatment (Figure 5B). The HFD group exhibited a significant increase in both HDL‐c (Figure 5C) and LDL‐c (Figure 5D) compared to NFD. While YNG‐H significantly reduced HDL‐c levels, both YNG‐L and YNG‐H treatments resulted in a significant increase in LDL‐c levels compared to the HFD group.

#### 3.2.4 Microscopic and Histological Analysis

**FIGURE 6:**
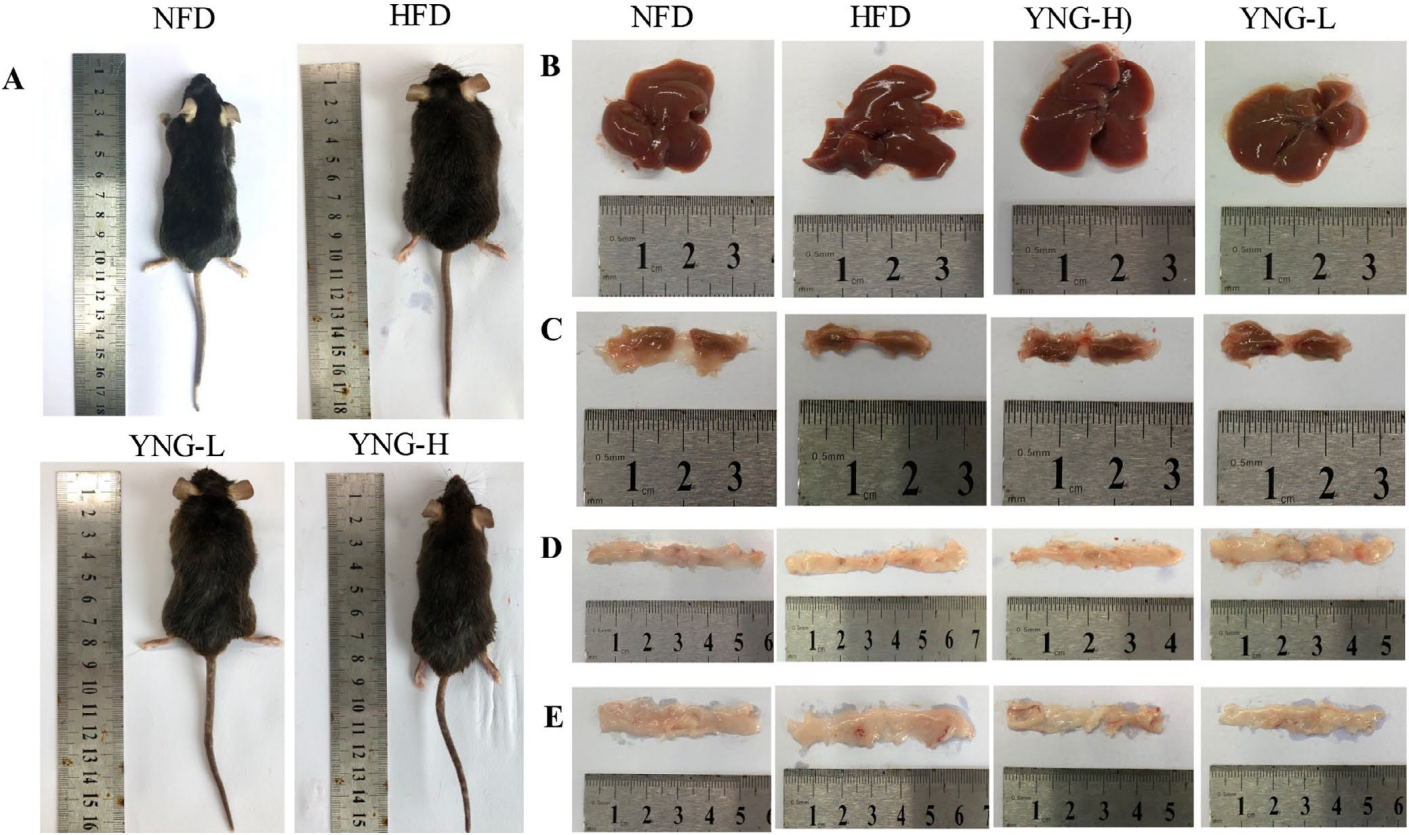
The microscopy of mice's liver tissues after 8 weeks of different diet treatments. (A) mice shape; (B) liver tissue morphology; (C) BAT tissue morphology; (D) IWAT tissue morphology; (E) EWAT tissue morphology. The morphology photos showed the effect of YNG extract on HFD mice. Compared with the NFD group, the HFD group had significantly enlarged body size and decreased body size after administration of YNG extract in the YNG‐L and YNG‐H groups. As to the tissue morphology, the tissue volume of liver, IWAT, and EWAT was enlarged in the HFD group compared with the NFD group and reduced dramatically in the YNG‐L and YNG‐H groups. Furthermore, the BAT tissue demonstrated the opposite trend with volume decreased in the HFD group and increased in the YNG‐L group.

**FIGURE 7:**
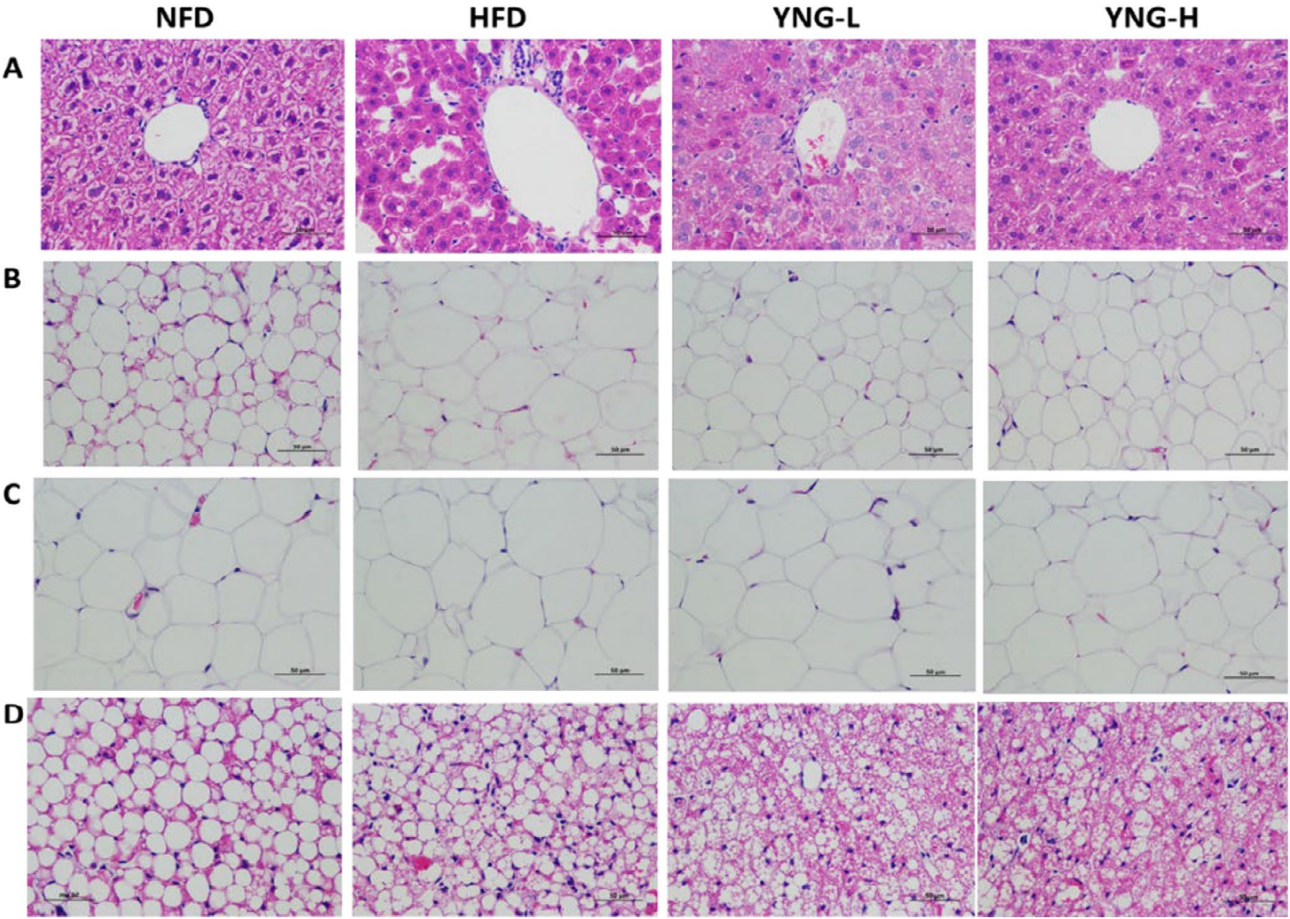
Effect of YNG‐based diet on microscopic changes as observed by hematoxylin and eosin staining images of the (A) liver tissues, (B) IWET tissues, (C) EWAT tissues, and (D) BAT tissues of mice. Magnification: 400×; Scale bar: 100 μm. BAT, brown adipose tissue; EWAT, epididymal white adipose tissue; HFD, high‐fat diet; IWAT, intra‐abdominal white adipose tissue; NFD, normal fat diet; YNG‐H, HFD receiving 300 mg/kg/day of *E. conferta* leaf extract; YNG‐L, HFD receiving 150 mg/kg/day of *E. conferta* leaf extract.

Hematoxylin and eosin (H&E) staining was performed to assess histological changes in liver and adipose tissues after 8 weeks of different dietary treatments (Figures 6 and 7). The body size of the HFD group was significantly enlarged compared to the NFD group. In contrast, administration of YNG extract in both YNG‐L and YNG‐H groups reduced body size (Figure 6A). Despite these overall differences, the liver morphology (Figure 6B) showed no significant macroscopic changes between the HFD and NFD groups, consistent with the reviewer's observation.

In contrast, substantial differences were observed in adipose tissues. The HFD group exhibited increased IWAT and EWAT volumes (Figure 6C,D) compared to the NFD group, indicating excessive fat accumulation. However, YNG treatment (YNG‐L and YNG‐H) led to a marked reduction in IWAT and EWAT volumes, suggesting a potential regulatory effect of *E. conferta* extract on fat storage. Additionally, BAT volume (Figure 6E) was reduced in the HFD group, which may indicate impaired thermogenic activity. Interestingly, BAT volume increased in the YNG‐L group, suggesting a potential enhancement of thermogenic function following YNG supplementation.

The liver and adipose tissue histology (Figure 7) further supported these macroscopic observations. The liver tissues of the HFD group exhibited increased fat vacuolation (Figure 7A), characterized by large intracellular lipid droplets indicative of hepatic lipid accumulation and steatosis. In contrast, the NFD group displayed a more uniform hepatocyte structure with minimal vacuolation. Notably, the YNG‐treated groups (YNG‐L and YNG‐H) demonstrated reduced vacuolation, suggesting that *E. conferta* extract mitigates HFD‐induced hepatic lipid accumulation.

Similarly, histological changes in adipose tissues aligned with the macroscopic findings from Figure 6. The HFD group exhibited enlarged adipocytes in IWAT (Figure 7B) and EWAT (Figure 7C), reflecting excessive lipid accumulation. YNG treatment significantly reduced adipocyte size, suggesting improved fat metabolism. In contrast to WATs, BAT volume was reduced in the HFD group (Figure 7D), consistent with macroscopic observations in Figure 6E. However, BAT from the YNG‐treated groups exhibited smaller lipid droplets, indicating enhanced BAT function and potential thermogenic activation.

## 4 Discussion

### 4.1 Comprehensive Phytochemical Profiling and Identification of Bioactive Metabolites in *E. conferta* Using UPLC‐Q‐TOF‐MS/MS

UPLC‐Q‐TOF‐MS/MS is a highly efficient multi‐component analytical technology that enables rapid and precise identification of plant metabolites (Joo et al. 2020; Wu et al. 2019). This method is distinguished by its broad scanning range, outstanding separation capability, high sensitivity, and mass accuracy, making it an essential tool for comprehensive phytochemical profiling (Joo et al. 2020; Wu et al. 2019). The application of UPLC‐Q‐TOF‐MS/MS has significantly advanced the chemical characterization of plant extracts, facilitating the identification of various bioactive compounds, including flavonoids and phenolics (Khan and Ali 2015; Yang et al. 2023). Moreover, its ability to differentiate metabolites based on unique mass spectra and retention times enhances the accuracy of compound identification, particularly in complex plant matrices. Given the structural similarities often observed among phytochemicals, the high‐resolution capabilities of this technique are crucial for distinguishing between structurally related compounds, thereby improving the reliability of metabolite identification (Khan and Ali 2015; Yang et al. 2023).

In this study, the methanolic extract of *E. conferta* leaves was analyzed using UPLC‐Q‐TOF‐MS/MS in negative ionization mode, as this mode demonstrated superior ionization efficiency and improved flavone sensitivity compared to positive ionization. As a result, 13 distinct secondary metabolites were tentatively identified, including eight flavones, three phenolic acids, one sugar derivative, and one organic sugar acid, underscoring the chemical diversity of *E. conferta* . Among these, daucic acid, a sugar acid, has been detected in various plant species and is known for its potential biological activity (Keys et al. 1971). Similarly, D‐glucaro‐1,4‐lactone, a sugar derivative, has been associated with oxidative and detoxifying properties, with reported anticancer effects (Olas et al. 2007; Saluk‐Juszczak et al. 2008).

The identified phenolic derivatives, including caffeic acid 3‐O‐glucuronide, 3‐glucosyl‐2',4',6'‐trihydroxy acetophenone, and 1‐O‐feruloylglucose, contribute to the extract's potential health benefits due to their well‐documented antioxidant and anti‐inflammatory properties (Abulizi et al. 2019; Ghaben and Scherer 2019; Gill and Gupta 2018; Patil et al. 2012; Valvi et al. 2014). Likewise, flavones such as aromadendrin 3,7‐diglucoside, calendoflaside, rutin, ranupenin 3‐rutinoside, kaempferol 3‐neohesperidoside, keioside, spinacetin 3‐gentiobioside, and tamarixetin are widely recognized for their metabolic regulatory effects, including lipid metabolism modulation and anti‐adipogenic activity (Gupta et al. 2021; Patil et al. 2012; Valvi et al. 2014). These findings suggest that *E. conferta* possesses a diverse phytochemical composition with potential therapeutic applications (Gupta et al. 2021; Patil et al. 2012; Valvi et al. 2014).

A more in‐depth pharmacological assessment of these metabolites is crucial to establishing their specific biological functions, particularly in relation to obesity management. The identification of flavonoids and phenolic acids in *E. conferta* enhances our understanding of its bioactive potential and paves the way for further research into its application in drug development. These compounds have been extensively studied for their antioxidant, anti‐inflammatory, and antimicrobial properties, reinforcing the medicinal significance of *E. conferta* (Gupta et al. 2021; Patil et al. 2012; Valvi et al. 2014; Yang et al. 2023). The comprehensive characterization of its phytochemical profile not only strengthens its pharmacological relevance but also supports its potential use as a natural therapeutic agent for metabolic disorders.

Overall, the integration of UPLC‐Q‐TOF‐MS/MS in the phytochemical analysis of *E. conferta* represents a significant advancement in plant metabolomics. The ability of this technique to accurately detect and differentiate metabolites highlights its critical role in natural product research. Continued application of high‐resolution mass spectrometry will further expand our understanding of plant‐derived compounds, facilitating their pharmaceutical, nutraceutical, and functional food applications.

### 4.2 In Vivo Anti‐Obesity Activity of *E. conferta* Leaves Extract

The biological properties of *Elaeagnus* species include analgesic and antidotal effects for various ailments, such as asthma, cancer, ulcers, and arthritis (Gupta et al. 2021; Valvi et al. 2014). Additionally, these species have demonstrated anti‐bacterial, anti‐ulcerogenic, and anti‐diabetic properties (Abulizi et al. 2019; Ghaben and Scherer 2019; Gill and Gupta 2018; Gupta et al. 2021; Lu et al. 2020).

This study uses a murine model to build upon existing knowledge by exploring the in vivo anti‐obesity effects of *E. conferta* leaf extract (YNG). Results revealed that mice on a HFD exhibited a significant increase in body weight compared to that on a NFD, which was accompanied by increased fat accumulation in the liver and adipose tissues. The excessive energy intake from the HFD resulted in fat deposition, with elevated IWAT and EWAT volumes (Figure 6C,D). This increase aligns with known mechanisms of obesity, where surplus calories promote TG storage, adipocyte hypertrophy, and WAT expansion (Abulizi et al. 2019; Bais and Patel 2020; Ghaben and Scherer 2019; Hu et al. 2022; Lu et al. 2020).

Macroscopic examination (Figure 6) further supports these findings. While liver morphology (Figure 6B) did not exhibit significant differences between the HFD and NFD groups, consistent with the reviewer's observation, histological analysis (Figure 7A) revealed pronounced hepatic lipid vacuolation in the HFD group, suggesting early‐stage steatosis (VanSaun et al. 2009). This indicates that hepatic fat accumulation may not always be apparent macroscopically but can be detected at the histological level. The liver weight was also significantly higher in the HFD group, supporting the idea that lipid overload occurs despite no gross morphological changes.

Notably, YNG administration significantly counteracted these effects, demonstrating a dose‐dependent effect on weight reduction (Han et al. 2022). Mice supplemented with YNG‐L and YNG‐H showed marked decreases in body weight, liver weight, and adipose tissue mass (Figure 4) suggesting that *E. conferta* extract effectively mitigates HFD‐induced lipid accumulation (Fan et al. 2021; Han et al. 2022). IWAT and EWAT volumes (Figure 6C,D) in the treated groups highlight the potential of YNG extract in suppressing adipogenesis and lipid storage in WAT. This aligns with studies suggesting that plant‐derived bioactive compounds can modulate lipid metabolism, enhance lipolysis, and promote adipose browning (Bais and Patel 2020; Fan et al. 2021; Han et al. 2022; Hu et al. 2022; VanSaun et al. 2009).

Further supporting this, BAT volume (Figure 6E) was significantly reduced in the HFD group, a key indicator of impaired thermogenic activity. However, YNG supplementation restored BAT volume, particularly in the YNG‐L group, suggesting that *E. conferta* extract may enhance thermogenesis and energy expenditure (Reguero et al. 2021). This aligns with Figure 7D, where BAT in YNG‐treated mice displayed smaller lipid inclusions, indicating enhanced lipid utilization and thermogenic activation (Fan et al. 2021; Reguero et al. 2021).

Biochemical analysis further reinforced these effects, showing reduced blood TG, TC, and LDL levels, along with increased HDL levels in YNG‐treated groups (Figure 5). These changes are indicative of improved lipid metabolism, as HFD‐induced obesity is often accompanied by dyslipidemia (Hu et al. 2022). Additionally, the decreased liver weight in YNG‐treated mice suggests that *E. conferta* extract mitigates hepatic lipid accumulation, which is a common consequence of excessive fat intake (Burcelin et al. 2002; Munkong et al. 2024; Nderitu et al. 2023).

The mechanisms underlying these anti‐obesity effects likely stem from the rich phytochemical profile of *E. conferta* . UPLC‐Q‐TOF‐MS analysis identified 13 distinct secondary metabolites, including flavones and phenolic acids, each known to influence lipid metabolism and adipogenesis (Bais and Patel 2020; Han et al. 2022; VanSaun et al. 2009). Several flavones, including rutin, kaempferol, and spinacetin, have been extensively studied for their ability to reduce fat accumulation, enhance lipolysis, and improve metabolic efficiency (Liu et al. 2023; Wang et al. 2022; Xu et al. 2020). Additionally, phenolic acids, such as caffeic acid, have been reported to modulate lipid metabolism, suppress adipogenesis, and exhibit antioxidant properties (Gutierrez et al. 2019; Xu et al. 2020; Zang et al. 2015). These bioactive compounds may explain the observed reductions in WAT mass, hepatic fat, and overall body weight in YNG‐treated groups.

In summary, *E. conferta* leaf extract presents a promising natural intervention for obesity, exhibiting strong potential to regulate lipid metabolism, enhance thermogenic activation, and suppress adipocyte hypertrophy. The macroscopic and histological findings from Figures 6 and 7 provide consistent evidence that YNG extract reduces WAT storage while promoting BAT activity. These findings support previous reports that plant polyphenols and flavonoids can combat obesity by modulating adipogenesis, thermogenesis, and lipid homeostasis (Gutierrez et al. 2019; Liu et al. 2023; Reguero et al. 2021; Wang et al. 2022; Xu et al. 2020; Zang et al. 2015).

While our findings highlight the anti‐obesity potential of *E. conferta* extract, further research is needed to clarify its molecular mechanisms, particularly its role in thermogenesis, adipocyte differentiation, and metabolic pathways (e.g., AMPK, PPARγ, and UCP1). Additionally, studies on its long‐term effects and safety are essential to establish its clinical relevance for obesity management.

## 5 Conclusions

Obesity is a major health concern, necessitating safer and more effective therapeutic interventions. Our study provides the first evidence of the anti‐obesity potential of *E. conferta* leaf extract, demonstrating its ability to reduce weight gain, lower LDL and TC levels, and decrease adipocyte size in HFD‐induced obese mice. Additionally, the extract mitigated hepatic and adipose lipid accumulation, suggesting its role in regulating lipid metabolism. These findings highlight *E. conferta* as a promising natural candidate for obesity management. Further research is needed to elucidate its molecular mechanisms and assess its efficacy in human studies for potential therapeutic applications.

## Author Contributions

Conceptualization: J.Z., P.W., H.Y., X.Z., Y.H., M.A.E., Y.L., W.M., M.F., and J.W.; methodology: J.Z., P.W., Y.L., W.M., and J.W.; software: M.A.E.; validation: W.M., M.F., and J.W.; formal analysis: J.Z., P.W., Y.H., X.Z., and H.Y., investigation: J.Z., P.W., X.Z., M.A.E., W.M., M.F., and J.W.; resources: J.W.; data curation: W.M., M.F., and J.W.; writing – original draft preparation: J.Z., P.W., X.Z., M.A.E., Y.L., W.M., and J.W.; writing – review and editing: J.Z., P.W., Y.H., M.A.E., Y.L., W.M., and J.W.; funding acquisition: M.F. and J.W. All authors have read and agreed to the published version of the manuscript.

## Disclosure

Institutional Review Board Statement: Animal procedures were performed according to the Guidelines for Animal Care and Use Committee of the Chinese Academy of Tropical Agricultural Sciences (ACUCC), Hainan, PR China.

## Conflicts of Interest

The authors declare no conflicts of interest.

## Data Availability

All the data are included in the article.
